# Effects of Storage Temperature at the Early Postharvest Stage on the Firmness, Bioactive Substances, and Amino Acid Compositions of Chili Pepper (*Capsicum annuum* L.)

**DOI:** 10.3390/metabo13070820

**Published:** 2023-07-05

**Authors:** Yuan Cheng, Chengan Gao, Shaodan Luo, Zhuping Yao, Qingjing Ye, Hongjian Wan, Guozhi Zhou, Chaochao Liu

**Affiliations:** 1 State Key Laboratory for Managing Biotic and Chemical Threats to the Quality and Safety of Agro-Products, Institute of Vegetables, Zhejiang Academy of Agricultural Sciences, Hangzhou 310021, China; chengyuan@zaas.ac.cn (Y.C.); yaozp@zaas.ac.cn (Z.Y.); yeqj@zaas.ac.cn (Q.Y.); hjwan@zaas.ac.cn (H.W.); zhougz@zaas.ac.cn (G.Z.); 2 College of Biotechnology, Jiangsu University of Science and Technology, Zhenjiang 212018, China

**Keywords:** pepper fruit, postharvest quality, temperature, capsaicin, amino acid composition

## Abstract

The commercial and nutritional quality of chili peppers deteriorates rapidly after harvest. So far, little is known about the effect of temperature on postharvest chili pepper quality. This study elucidated the effects of two temperatures (20 °C and 30 °C) on chili peppers’ postharvest firmness, flavor, and nutritional attributes. We found that compared to 20 °C, 30 °C escalated the decline in fruit firmness, capsaicin content, and dihydrocapsaicin content, while enhancing the increment in water loss and electrical conductivity, as well as total carotenoids and ascorbic acid content. The contents of most amino acids (AAs) decreased significantly during postharvest storage compared to their initial values, whether stored at 20 °C or 30 °C; however, 30 °C had a more substantial impact than 20 °C. Meanwhile, as for soluble protein and amino acid compositions, the effect of storage temperature was genotype-dependent, as reflected by differential changes in total AA contents, single AA contents, essential AA ratio, delicious AA ratio, etc., under the 20 °C or 30 °C treatments. In conclusion, our findings reveal the influence of temperature on pepper quality, showing that the storage temperature of 20 °C was better for maintaining chili quality than 30 °C from the perspective of overall commercial attributes.

## 1. Introduction

Chili pepper (*Capsicum annuum* L.) is one of the most important spice and vegetable crops in the world [1,2]. Pepper fruits are rich in bioactive substances, including capsaicinoids, vitamin C (ascorbic acid), pigment substances, amino acids, etc. [3]. Usually, the quality of pepper fruit is determined by its commodity, flavor, and nutritional value [3,4,5]. Capsaicinoids are a group of alkaloids that give appealing pungency to the chili pepper of the *Capsicum* genus. Capsaicin and dihydrocapsaicin constitute more than 80% of capsaicinoids, depending on genotypes [1,6]. In addition to the pungent flavor, capsaicinoids have also been demonstrated to exhibit antioxidant, anticarcinogenic, and thermogenic properties [7,8,9]. In addition to capsaicinoids, different antioxidant substances (such as chlorophyll, ascorbic acid, carotenoids, etc.) are well known for their health benefits, such as anti-aging effects and reducing the risk of cancer [3,5,10]. Amino acids are essential nutrients for the growth and health of humans [11], and the composition and contents of amino acids also affect the nutritional quality and taste of vegetables [12,13].

Due to their high moisture content (70–90%), postharvest pepper fruits usually undergo vigorous water loss and other metabolic activities, resulting in fruit softening, degradation of biologically active substances, and the failure of nutritional benefits. These factors considerably impact shelf life, consumer preference, and transportability, making it challenging to maintain the quality of the product [14,15]. Proper storage temperature has been demonstrated to be critical for maintaining postharvest fruit quality [16,17,18,19]. According to a previous study, the excess temperature would accelerate the after-ripening process, along with water loss, wilting, and decay of harvested pepper fruits [16]. As described by Wei et al. [20], the storage process is accompanied by a decrease in chlorophyll contents and the accumulation of carotenoids, while up to 50% of carotenoids are capsanthin in peppers [16]. The effects of temperature on the ripening process and quality component vary depending on the crop [21,22,23,24]. For instance, compared to relatively low temperatures, storage under higher temperatures (30 ± 2 °C) would significantly accelerate the ripening, chlorophyll degradation, and total carotenoid accumulation in mango (*Mangifera indica*) [23], as well as promote water loss, fruit softening, and reduction in ascorbic acid content [24]. The storage experiments conducted on postharvest citrus (*Citrus reticulata*) and tomato (*Solanum lycopersicum*) fruits demonstrated that the lutein in citrus and lycopene in tomato both decreased under low temperature (5 °C) and high temperature (30 ± 2 °C) conditions, compared to the fruits preserved at room temperature [21,22]. However, the accumulation of zeaxanthin and β-carotenoids in citrus fruits increased under high temperatures. Some studies reported that the chlorophyll degradation and the accumulation of total carotenoids induced by unsuitable temperatures were attributed to the stress-inducing accumulation of reactive oxygen species (ROS), promoting the synthesis of antioxidant substances (capsaicinoids, carotenoids, ascorbic acid, etc.) for the elimination of ROS [25,26,27].

So far, most postharvest storage methods involving temperature control have been focused on the relatively low-temperature preservation of horticultural product organs, including pepper fruits [28,29,30]. Few studies have been carried out on the quality changes during short-term storage of peppers under natural environments. The harvest season of chili peppers in many planting areas of China usually lasts from April to July. Considering the market and warehouse costs comprehensively, the harvested chili peppers typically go through temporary storage for 0–2 days under a natural environment, with average temperatures ranging from 20 °C to 30 °C (depending on the picking month). Here, to thoroughly evaluate the quality changes in harvested pepper fruits under different temperatures, we investigated the effects of 20 °C and 30 °C on the fruit quality of three different pepper genotypes. The results deepen our understanding of the effect of temperature on postharvest pepper storage.

## 2. Materials and Methods

### 2.1. Material Collection and Temperature Treatment

Three pepper cultivars P1632, P1833, and P1622 (*C. annuum*), newly bred by the Zhejiang Academy of Agricultural Sciences (ZAAS, Hangzhou, China) and each displaying a distinct phenotype (Table 1), were chosen for experiments in the present study. P1632, P1833, and P1622 are three representative pepper types that are widely consumed in different areas of China. The experiments were carried out at the experimental farm of the ZAAS (longitude 120°2′ E, latitude 30°27′ N), Zhejiang Province, China, during the April to July 2019 growing season. Seedlings of the 5-true-leaf stage were transplanted into the field (30 cm × 60 cm), and they received regular irrigation and fertilization management. Flowers were tagged to mark the anthesis date, and pepper fruits of the 30th day post-anthesis (DPA) of each cultivar were picked up for further temperature treatment.

Ninety-six fruits each of P1632, P1833, and P1622 (288 fruits in total) were selected and divided into three groups, with one group acting as a control, which was used to determine the initial values of all of the parameters that we detected in this study before exposure to temperature treatments, while the other two groups were exposed to 20 °C (representing the natural temperature of April) and 30 °C (representing the higher temperature of July), respectively. The experimental conditions were dark and with 85% relative humidity. After firmness measurement, three biological replicates, each consisting of six fruits (n = 6), were frozen with liquid nitrogen and stored at −80 °C for further analysis.

### 2.2. Morphological and Firmness Determination

The plant height, canopy width, and fruit length were measured using a flexible ruler, and the fruit diameter and single fruit weight were tested with Vernier calipers (Mitutoyo505–732, Tuofeng instrumental Ltd., Nanjing, China) and an electronic balance (HZY-B320, Yajing instrumental Ltd., Shanghai, China), respectively. The fruit number of each plant was manually counted, and the fruit yield of each plant was calculated by single fruit weight × fruit number plant^−1^. Ten biological replicates were tested for the parameters mentioned above.

Fruit firmness was tested on the middle region with a Model GY-3 texture analyzer (huaibo Instrument Equipment Co., Ltd., Xuzhou, China) and a metal probe of 10 mm in diameter. The penetration depth was 15 mm. The unit of measurement for firmness is N.

### 2.3. Water Loss Rate and Relative Electrolyte Leakage

The fresh weight of each fruit was weighed and recorded at each time point (0 h, 24 h, and 48 h). The following formula was used to calculate the weight loss rate: weight loss rate (%) = (the initial fruit weight − the final fruit weight)/the initial fruit weight × 100.

Nine randomly selected fruits from each treatment group were used to determine the relative electrolyte leakage, with three fruits from each biological replicate used. Ten slices with a uniform thickness of approximately 0.8 mm were cut from the middle of each pepper fruit. The slices were rinsed with deionized water three times and placed into a 50 mL tube with 20 mL of deionized water, followed by shaking at 200 rpm in a shaker at 25 °C for one hour. The initial electrical conductivity (EC1) was measured with a digital electrical conductivity meter (DDS-307 A, Inesa Inst. Co., Shanghai, China). Afterward, each sample was boiled in a water bath for 10 min and cooled to room temperature with running water; then, the final electrical conductivity (EC0) was determined, and the REL was calculated with the equation in percentage: REL (%)  =  EC_1_/EC_0_ × 100.

### 2.4. HPLC Analysis of Capsaicinoids and Ascorbic Acid

Capsaicinoids, including capsaicin and dihydrocapsaicin, were extracted and quantified according to the previously described method, with some modifications [31]. Briefly, frozen fruit tissues were ground with a mortar and pestle, and 10 g of fresh homogenate of each sample was lyophilized for 48 h. Dehydrated samples (1–1.5 g) were extracted with methanol in plastic tubes in a water bath at a temperature of 60 °C for 30 min, with manual agitation every 10 min, and followed by two repeated cycles. The collected extract solution was filtered twice with filter paper and centrifuged at 14,000× *g* for 15 min. The supernatant was collected and filtered through a Millipore membrane filter (0.45 μm pore size, Thermo Scientific, Shanghai, China). The filtrates were stored at −20 °C in dark bottles and used to separate and quantify capsaicinoids. Capsaicin and dihydrocapsaicin were separated using an HPLC system consisting of a Waters 600 separations module and a Waters 2414 RI detector (Waters Corp., Milford, MA, USA), using methanol/water (65/35, *v*/*v*) as the mobile phase. The flow rate was 1 mL min^−1^, and capsaicinoid detection was conducted at 280 nm. Capsaicin (97%) and dihydrocapsaicin (90%) standards were purchased from Sigma (St. Louis, MO, USA). Each sample was confirmed with three biological replicates. The capsaicinoids were expressed as mg kg^−1^ fresh weight (FW).

The extraction of ascorbic acid was carried out as described by Liu et al. [32], with some modifications. The freeze-dried fruit pericarps were ground into powder, and 0.2 g of each sample was extracted with an oxalic acid solution (1%, *w*/*v*). The extracts were centrifuged at 14,000× *g* for 10 min, and the collected supernatants were used for HPLC analysis. Samples were injected and separated in a C18 column (5 μm, 4.6 mm× 250 mm, Elite instrumental Ltd., Dalian, China) using 0.1% oxalic acid (*w*/*v*) as the mobile phase at a flow rate of 1 mL min^−1^. The ascorbic acid was calculated at the absorbance value of 243 nm, using authentic ascorbic acid (Sigma-Aldrich, Shanghai, China) as a standard. The ascorbic acid content was presented as mg kg^−1^ fresh weight (FW).

### 2.5. Soluble Protein Assay

The protein content was determined according to Bradford’s method, as previously reported [33], with certain modifications. First, 0.3 g of fresh sample (homogenate) was extracted with 5 mL of extraction buffer (200 mM Tris, 10 mM EDTA, pH 5.5). The protein dissolved in the supernatant was measured by Coomassie brilliant blue G-250 (Shenggong Ltd., Shanghai, China) and further calculated with the absorbance value at 595 nm using bovine serum albumin (Sigma-Aldrich, Shanghai, China) as a standard. The results were expressed as g kg^−1^ fresh weight (FW).

### 2.6. Total Carotenoids Assay

The total carotenoids were extracted and measured as described by Zhao et al. [33]. The homogenate samples were added to 3 mL of acetone, followed by ultrasonication treatment for extraction. The total carotenoids of each sample were separated using a Waters 600 HPLC system in a C18 column (4 μm, 3.9 mm × 150 mm, Elite instrumental Ltd., Dalian, China) and detected using a 2487 UV detector at 448 nm.

### 2.7. Amino Acid Detection

The fruit homogenate (100 mg) was placed in a 20 mL hydrolysis tube, and 10 mL of 6 M HCl was added. The tube was then evacuated with a vacuum pump, filled with high-purity nitrogen, and sealed. The sealed samples were then hydrolyzed at 110 °C for 24 h and dried at 55 °C in a vacuum desiccator. The dried samples were dissolved in 5 mL of sodium citrate buffer (pH 2.2) and filtered through a 0.22 μm membrane filter. Amino acid contents were determined using a Hitachi L8800 Amino Acid Analyzer (Hitachi High-Technologies Corporation, Tokyo, Japan) equipped with a 2620 MSC-PS column (80 × 4.6 mm). The peak spectrum of the resulting sample was compared to the standard amino acid spectrum. The amino acid contents in Table 2 and Table S1 are expressed in g kg^−1^ FW. As for the amino acid nutrition valuation in Table 3, the percentage of each human essential amino acid in the total amino acids was calculated and compared with the amino acid pattern spectrum reported by FAO/WHO/UNU [11].

### 2.8. Statistical Analysis

Data were expressed as the mean ± standard error of three replicates. Statistical analysis of the data was performed with SPSS Version 19.0 (SPSS Inc., Chicago, IL, USA). Data were tested for significant (*p* < 0.05) treatment differences using one-way ANOVA, followed by Tukey’s test.

## 3. Results

### 3.1. Effects of Storage Temperature on the Contents of Capsaicinoids

**Figure 1:**
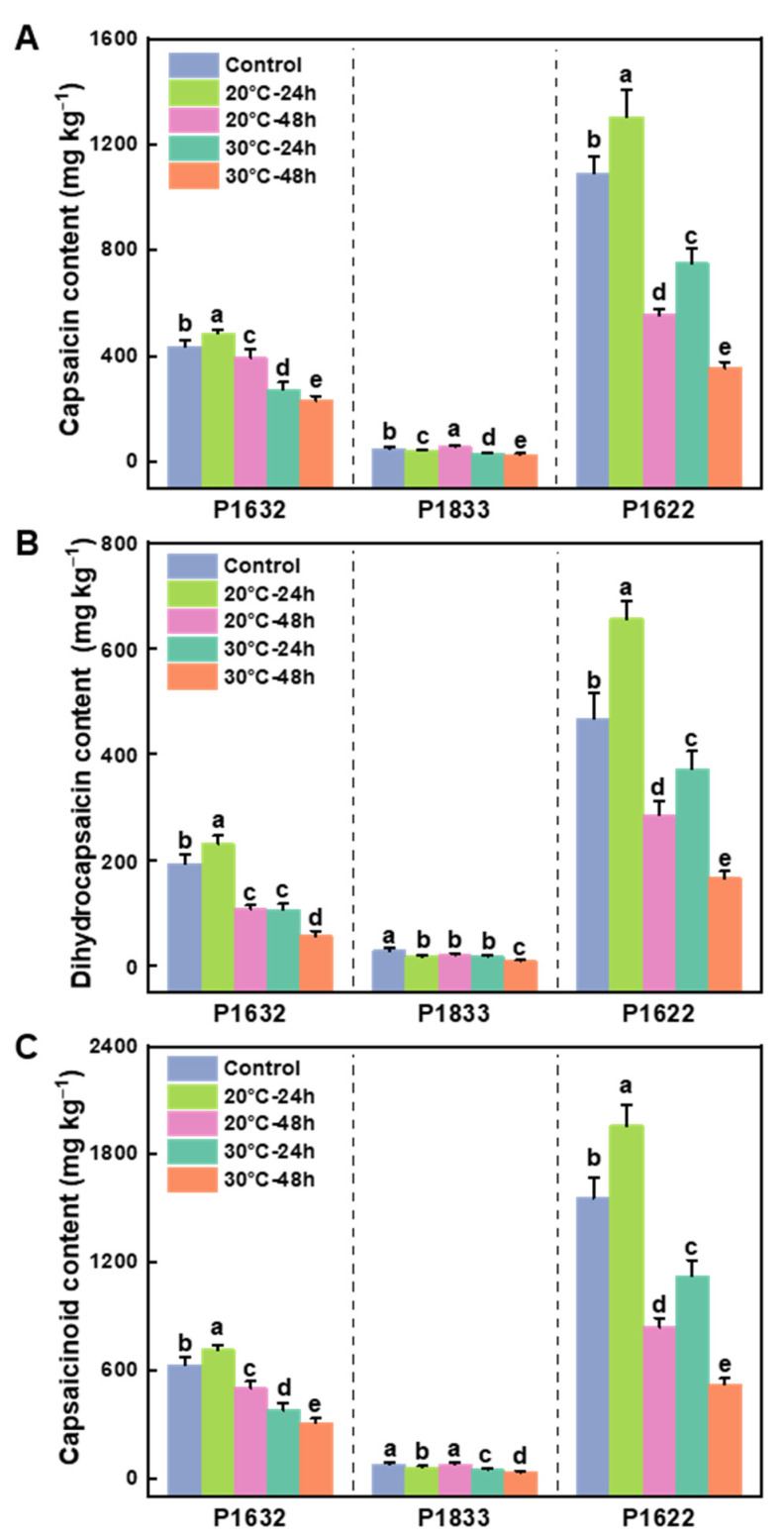
Effects of storage temperature on capsaicin (**A**), dihydrocapsaicin (**B**), and capsaicinoid (**C**) contents in pepper fruits of three cultivars. Results are shown as the mean ± SE of three biological replicates (for each replicate, n = 6). Means denoted by the same letter were not significantly different at *p* < 0.05 according to Tukey’s test. Control represents the initial values of fruits before being exposed to the temperature treatments, with 20 °C simulating the temperature of April and 30 °C simulating the temperature of July.

The results showed that 30 °C resulted in a sustained and severe reduction in capsaicin and dihydrocapsaicin contents, as well as the total capsaicinoid content, in all three cultivars as the storage duration progressed (Figure 1). Specifically, under 30 °C conditions, the capsaicin content decreased by 31–37% in all three cultivars at 24 h, but further reduced by 42–47% in P1632 and P1833 and by 67% in P1622 at 48 h (Figure 1A). Meanwhile, at 24 h, dihydrocapsaicin decreased by 44% in P1632, 36% in P1833, and only 20% in P1622; however, at 48 h, the capsaicin content was severely reduced by 64–70% in all three cultivars (Figure 1A,B). Interestingly, for the fruits of P1632 and P1622 stored at 20 °C, there was a significant increment for both capsaicin and dihydrocapsaicin contents at 24 h, but this was followed by a severe reduction at 48 h, much as occurred for the fruits treated at 30 °C. No matter whether at 24 h or 48 h, both the capsaicin and dihydrocapsaicin contents decreased to a greater extent in the fruits treated at 30 °C than in those treated at 20 °C for all three cultivars, except for P1833 at 24 h, in which the dihydrocapsaicin content of the fruits treated at 30 °C was same as that of those stored at 20 °C (Figure 1A, B). Consequently, the contents of capsaicinoids, which are mainly composed of capsaicin and dihydrocapsaicin, showed similar change trends to that of capsaicin (Figure 1C).

### 3.2. Effects of Storage Temperature on Fruit Firmness, Water Loss Rate, and Relative Electrolyte Leakage

**Figure 2:**
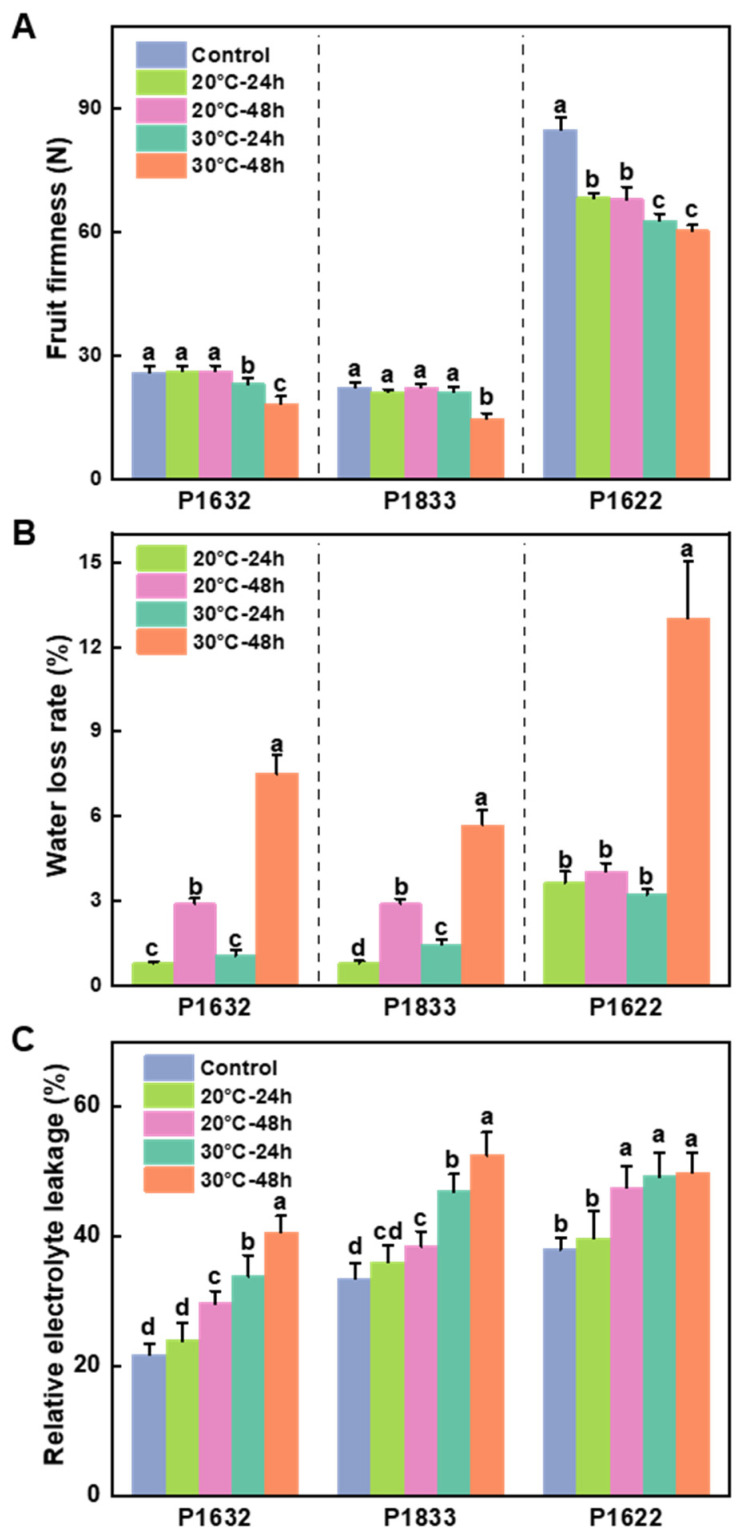
Effects of storage temperature on fruit firmness (**A**), water loss rate (**B**), and relative electrolyte leakage (**C**). Results are shown as the mean ± SE of 32 samples for fruit firmness (**A**) and water loss rate (**B**), while 9 samples were used for relative electrolyte leakage (**C**). Means denoted by the same letter were not significantly different at *p* < 0.05 according to Tukey’s test. Control represents the initial values of fruit before being exposed to the temperature treatments, with 20 °C simulating the temperature of April and 30 °C simulating the temperature of July.

For fruits stored at 20 °C, we found that the fruit firmness of P1632 and P1833 was not affected either at 24 h or 48 h, but the fruit firmness of P1622 decreased significantly by 19% at 24 h and remained the same at 48 h. When stored at 30 °C, the fruit firmness of P1632 and P1622 reduced considerably—by 11% and 26%, respectively—but the fruit firmness of P1833 remained the same as the control at 24 h. However, the fruit firmness of all three cultivars at 30 °C was severely decreased by 29–34% at 48 h (Figure 2A).

We found that no matter whether stored at 20 °C or 30 °C, there was a significant increase in WLR from 24 h to 48 h for all three cultivars, except for P1622 at 20 ℃. Notably, after storage for 48 h, the extent of the increment in the absolute value of WLR was much more significant for fruits stored at 30 °C than for those stored at 20 °C across the three cultivars. For instance, it increased by 251% at 20 °C versus 590% at 30 °C in P1632, and by 255% at 20 °C versus 283% at 30 °C in P1833. Similarly, there was no significant difference at 20 °C, while it increased by 300% at 30 °C in P1622. In addition, at 24 h, there was no difference between 20 °C and 30 °C for WLR in both P1632 and P1622; however, the WRL was higher at 30 °C than at 20 °C in P1833 (Figure 2B).

When stored at 20 °C for 24 h, the relative electrolyte leakage (REL) of all of the tested cultivars was still indistinguishable from the control. However, after 48 h at 20 °C, the REL of all of the cultivars increased significantly compared to that of both control and 24 h. For fruits stored at 30 °C, the REL of all of the cultivars increased significantly and continuously even at 24 h. Meanwhile, it is worth noting that the REL of fruits stored at 30 °C was much greater than that of those stored at 20 °C at each time point, except for P1622 at 48 h (Figure 2C).

### 3.3. Effects of Storage Temperature on the Nutrient Quality of Fruits

**Figure 3:**
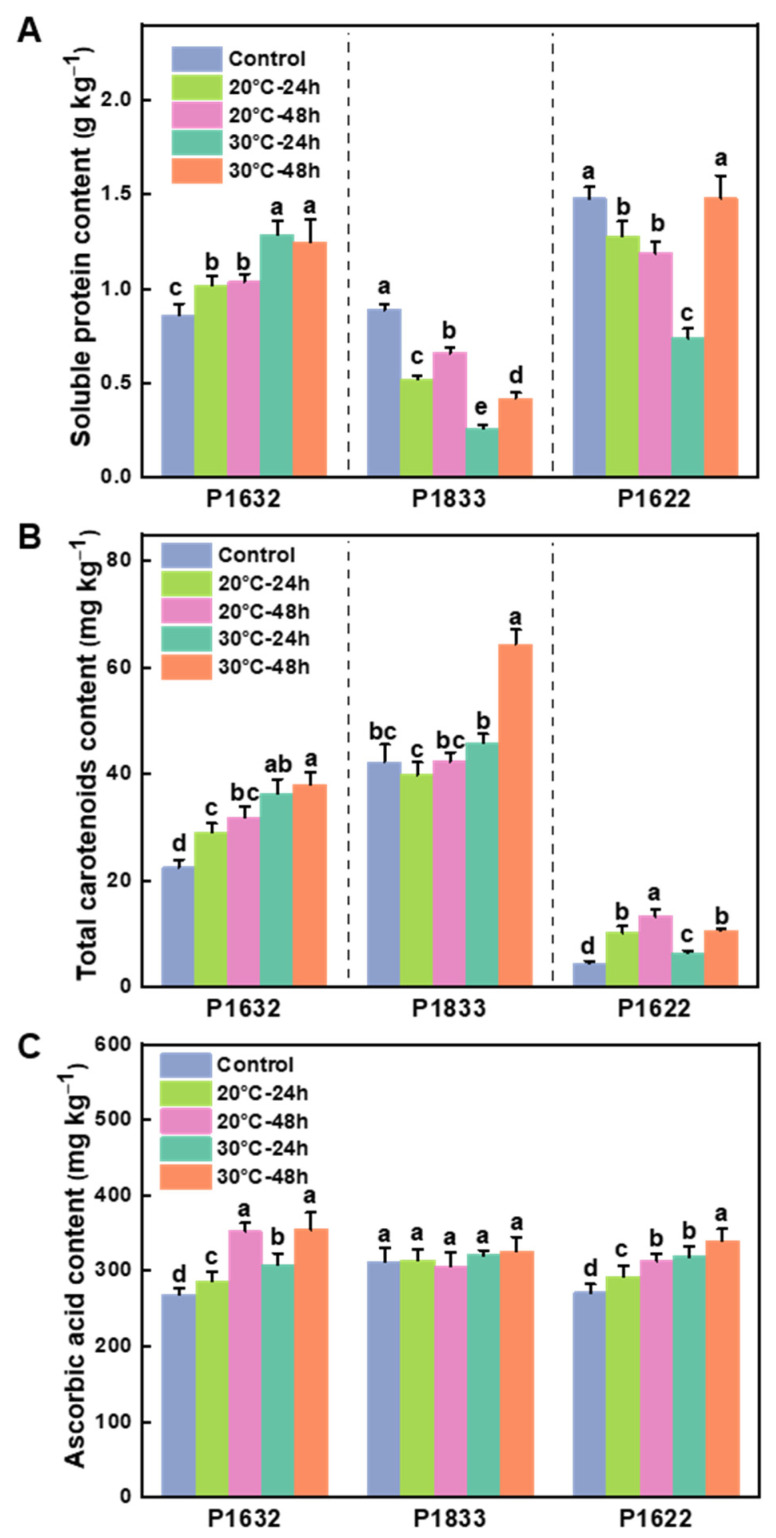
Effects of storage temperature on soluble protein content (**A**), total carotenoid contents (**B**), and ascorbic acid content (**C**). Results are shown as the mean ± SE of three biological replicates (for each replicate, n = 6). Means denoted by the same letter were not significantly different at *p* < 0.05 according to Tukey’s test. Control represents the initial values of fruit before being exposed to the temperature treatments, with 20 °C simulating the temperature of April and 30 °C simulating the temperature of July.

After 24 h, the protein content of P1632 increased significantly, by 18% at 20 °C and 50% at 30 °C, which remained unchanged until 48 h. Conversely, it decreased by 41.4% and 71% at 20 °C and 30 °C in P1833, respectively, and by 13.8% and 50% in P1622, respectively. In addition, there was a slight but significant increment at 48 h under both 20 °C and 30 °C conditions in P1833, and a tremendous increment after 48 h at 30 °C in P1622. The protein content decreased significantly compared to the control after 48 h at both 20 °C and 30 °C in P1833, and at 20 °C in P1622, while the protein content was restored to the same level as the control when stored at 30 °C in P1622 (Figure 3A).

As for the carotenoid content, it was shown that no matter whether stored at 20 °C or 30 °C, the carotenoid content generally increased as the storage went on, except that there was no significant change in P1833 at 20 °C. Notably, at each time point, the carotenoid content was higher in fruits stored at 30 °C than in those stored at 20 °C in P1632 and P1833, while it was lower when held at 30 °C than at 20 °C in P1622 (Figure 3B).

Interestingly, the ascorbic acid content showed a similar change trend to that of carotenoids. Specifically, the ascorbic acid content in P1632 and P1622 kept increasing from 24 h to 48 h at a rate of ~7–25% at both 20 °C and 30 °C, and notably, the increment at 30 °C was more significant than that at 20 °C. In addition, ascorbic acid in P1833 was not influenced by storage at either 20 °C or 30 °C within 48 h (Figure 3C).

### 3.4. Effects of Storage Temperature on the Amino Acid Composition and Contents

In this study, we identified 17 amino acids from pepper fruit pericarps, including 7 essential amino acids, 10 non-essential amino acids, 2 essential amino acids for children [11], 5 umami amino acids, and 2 aromatic amino acids (Table 2 and Table S1). We found that no matter whether stored at 20 °C or 30 °C, the contents of most amino acids decreased to different extents; however, the effects of 20 °C and 30 °C were different for each cultivar. Specifically, in P1632, 20 °C decreased the amino acid content to a greater extent than 30 °C, especially for Ala, which was reduced by 19.8% at 20 °C versus 10.8% at 30 °C, and Phe, which was reduced by 57.0% vs. 12.8%, as well as Gly (19.4% vs. 9.1%). However, unlike P1632, 30 ℃ caused a much greater decrease in amino acid content than 20 °C in P1833. For instance, Asp decreased by 47.9% at 30 °C, while Glu decreased by 29.6%, Pro by 29.9%, Tyr by 51.5%, and His by 40.1%; however, these amino acids were not even affected at 20 °C, or they insignificantly decreased by no more than 5% at the mean level. As for P1622, almost all of these amino acids (13/17) decreased by 8–13% at both 20 °C and 30 °C, and interestingly, there was no difference between 20 °C and 30 °C. In addition, an exception was that Asp increased by 15.3% at 20 °C but decreased by 8.6% at 30 °C, while Cys increased by 20.0% at both 20 °C and 30 °C (Table S1).

We further analyzed the contents and composition ratios of different types of amino acids. As shown in Table 2, the total contents of amino acids decreased in all three cultivars at both 20 °C and 30 °C compared to their respective controls, decreasing by 22.2% at 20 °C versus 12.0% at 30 °C in P1632, and by 4.9% at 20 °C versus 28.0% at 30 °C in P1833. In contrast, there was no difference between 20 °C and 30 °C in P1622, with the same decrement of 8.6%. As for each specific amino acid type, we found that in P1632, aromatic amino acids decreased the most (by 40.1% at 20 °C versus 16.7% at 30 °C), followed by essential amino acids (by 26.3% at 20 °C versus 14.9% at 30 °C), and the rest by 18.9–20.3% at 20 °C versus 6.2–14.7% at 30 °C. Similarly, in P1833, aromatic amino acids and tasty amino acids decreased the most—by 36.7% and 36.1% at 30 °C, respectively—but they were not affected at 20 °C. Moreover, the other types of amino acid decreased by 23.4–31.1% at 30 °C but only by 3.1–7.4% at 20 °C. In P1622, except for the tasty amino acids, which were not affected at 20 °C but decreased by 5.7% at 30 °C, the other types of amino acids fell similarly. Specifically, the aromatic amino acids were also the most affected amino acids, and in P1632 and P1833 they decreased by ~12%. Meanwhile, sweet amino acids and essential amino acids decreased by ~11%, and non-essential amino acids and children’s essential amino acids decreased by ~7%, at both 20 °C and 30 °C. 

Interestingly, we found that the composition ratios of specific types of amino acid were also changed during storage. For instance, except for an increment in the essential amino acids (A) at 30 °C and aromatic amino acids (F) at 20 °C in P1833, the composition ratio of the essential amino acids (A) and aromatic amino acids (F) decreased significantly in all three cultivars. Likewise, when stored at 20 °C, non-essential amino acids (B), children’s essential amino acids (C), and MSG-like amino acids (D) were not affected in all three cultivars, except for an increment in the non-essential amino acids (B) in P1632 and MSG-like amino acids (D) in P1622; however, it showed a genotype-dependent change when stored at 30 °C—for instance, B and D increased in P1632, but B and D decreased in P1833 and were not affected in P1622.

### 3.5. Effects of Storage Temperature on the Essential Amino Acid Percentages

In most cases, 20 °C did not affect the essential amino acid (EAA) patterns, except for the decrements in Phe+ Tyr in P1632 and P1622, Thr in P1833, and Leu in P1622, as well as the increments in Phe+ Tyr in P1833 and Met+ Cys in P1622. However, the effect of 30 °C was genotype-dependent, where most (5/7) of the EAAs decreased in P1632 but were not affected in P1622, and some even increased in P1833 (Table 3).

## 4. Discussion

Temperature not only plays a pivotal role in regulating the growth and development of plants but also affects the quality changes of both leafy and fruit vegetables [34,35]. To better understand the quality changes of chili pepper fruits temporarily stored under natural conditions during the harvest season of peppers in China, two natural environmental temperatures (20 °C and 30 °C) were simulated for elucidating the effects of different temperatures on the postharvest quality preservation of peppers.

Capsaicin and dihydrocapsaicin (alkaloids) are two major pungent substances that determine the commercial quality of peppers, and their accumulation levels are mainly dependent on genotypes and various environmental factors, including light, soil moisture, and temperature [1]. Nevertheless, the effect of temperature on the capsaicinoid contents in peppers has not been well studied, especially for postharvest peppers. In this study, we found that the contents of capsaicin, dihydrocapsaicin, and total capsaicinoids continuously declined with the prolongation of the storage period, decreasing to a greater extent for the fruits stored at 30 °C than for those stored at 20 °C for all three tested pepper genotypes (Figure 1). Previously, we reported that high-temperature (36 °C) treatment reduced the capsaicinoid contents of pre-harvested pepper fruits by decreasing the transcription and corresponding protein abundance of capsaicin-biosynthesis-related genes [36]. Taking this into account, it could be speculated that the capsaicinoids may share similar metabolic mechanisms of high-temperature responses in both pre- and postharvest pepper fruits. In other studies, the accumulation patterns of capsaicinoids under high temperatures were reported to be genotype-dependent, as Gonzalez-Zamora et al. [37] found that the long-term high-temperature growing environment increased the capsaicinoid contents of five of the seven total tested varieties, while it decreased the capsaicinoid contents of the other two varieties. Based on this finding, we can suppose that different capsaicinoid accumulation modes might be found in the other genotypes of postharvest pepper fruits that we did not test in this study. Except for biosynthesis, it is well known that capsaicinoids can be oxidized by peroxidase in the presence of H_2_O_2_, and peroxidase can be activated when plants are challenged with stress [6,36,38]. It is thus indicated that as the storage duration progresses, the increasing activity of peroxidase may occur and may also play a certain role in diminishing the contents of capsaicin and dihydrocapsaicin. In this context, we only clarified the different change modes of capsaicinoid contents under different temperatures, but the regulation mechanisms need more detailed experimental confirmation, such as the capsaicin biosynthesis genes’ expression and the detection of peroxidase activity.

Fruit firmness is a critical fruit quality attribute that reflects the pulp’s resistance to pressure, which influences shelf life, consumer preference, and transportability [15]. During storage, fruit firmness reduction has been positively associated with weight loss, resulting from water loss driven by active metabolic processes, including transpiration and respiration in the fruit [39,40]. In the current study, we also observed that fruit stored at 30 °C exhibited a higher water loss rate and lower firmness. On the other hand, cell wall structure, strength, and intercellular adhesion are the primary determinants of fruit firmness quality and are regulated by several enzymes, such as β-galactosidase (β-GAL) and pectin lyase (PL), which could be influenced by the postharvest environment [15,41]. Therefore, assessing the contents of cell wall components and the activities of related enzymes would provide more detailed information to comprehend the effect of temperature on fruit firmness quality.

Carotenoids and ascorbic acid are two important phytochemicals that have excellent scavenging activity for ROS in plants, and their contents are dynamically influenced by genetics, ripening progress, and environmental factors [25,26,27]. In this study, we found that short-term storage at both 20 °C and 30 °C could promote the accumulation of the total carotenoids and ascorbic acid in most cases (Figure 3), and this was in accordance with the research findings reported by Pola et al. [16], who also tested postharvest mature green chili named “Takanotsume”. Pola et al. found that the postharvest chili peppers stored at 30 °C accumulated more β-carotene and capsanthin, as well as total carotenoids and ascorbic acid, compared to those stored at 20 °C. Nevertheless, one exception in our study was observed, as for the variety P1622 the total carotenoid content was higher when stored at 20 °C compared to 30 °C. These results reminded us that, like capsaicinoids, genotype should also be taken into consideration when studying the pigment compound mechanisms under different storage temperatures. Moreover, carotenoids are composed of many polyisoprenoid compounds, and they can be divided into two main groups: carotenes and xanthophylls, with β-carotene and capsanthin, respectively, being the dominant components of these two groups in pepper fruits [16,42]. Hence, a targeted metabolomic analysis in the future would be needed for more detailed assessment of the effects of storage temperature on the carotenoid compositions of postharvest peppers.

Amino acid contents and composition are important indicators for judging food quality [12,13]. As important primary metabolites, amino acids participate in many biological activities, such as energy and carbohydrate metabolism, responses to biotic and abiotic stresses, and secondary metabolism [43,44]. We found that although the relative effects of 20 °C and 30 °C in different varieties were different, the contents of most amino acids decreased significantly with the increasing storage duration in all three tested varieties (Table 2 and Table S1), indicating that the storage time is a more crucial factor than storage temperature in affecting amino acid accumulation. After harvest, the amino acid catabolic pathways in plants were hastened to offer substrates for ATP production and, thereafter, sustain normal cellular function and integrity, as the energy source from leaf photosynthesis was blocked, which would ultimately lead to the corresponding decrement in amino acid contents [45]. For instance, the catabolism of branched-chain amino acids (Leu, Ile, and Val) could produce acetyl-CoA for energy metabolism [43]. Amino acids are also precursors of many secondary metabolites; for example, Phe is the precursor of many flavonoids, which are ubiquitous in the plant kingdom, and their biosynthetic pathways are often triggered in response to biotic or abiotic stresses. [46]. Taken together, the loss of amino acid contents and altered amino acid composition are perhaps general deterioration features of postharvest peppers. Therefore, a lower storage temperature, such as 8 °C [18], to reduce the metabolic activity of postharvest peppers would reduce this loss.

## 5. Conclusions

The storage temperature at an early stage, within 48 h postharvest, can significantly affect the quality attributes of chili peppers. Compared to 20 °C, pepper fruits stored at 30 °C showed accelerated fruit softening, which was also reflected by increased water loss rate and electrical conductivity. Meanwhile, storage at 30 °C enhanced the decrement in capsaicin and dihydrocapsaicin contents, as well as the contents of most amino acids, but increased the total carotenoids and ascorbic acid contents. Moreover, the effects of storage temperature on soluble protein and amino acid composition showed a genotype-dependent response. In conclusion, based on this study, if short-term storage under a natural environment is unavoidable, chili peppers harvested in the early spring season would have better commercial quality compared to those harvested in the late spring and early summer.

## Figures and Tables

**Table 1:** Phenotypic characteristics of the three tested pepper cultivars.

Cultivar	Plant Height (cm)	Canopy Width (cm)	Fruit Length (mm)	Fruit Diameter (mm)	Single Fruit Weight (g)	Fruit Number Plant^−1^
P1632	49.25 ± 2.50 b	66.75 ± 3.59 b	135.13 ± 7.70 a	26.17 ± 1.55 a	29.65 ± 1.54 a	60.25 ± 11.87 b
P1833	75.50 ± 2.89 a	69.50 ± 6.03 ab	103.87 ± 1.36 b	24.74 ± 1.25 a	20.61 ± 1.65 b	22.50 ± 3.32 c
P1622	44.75 ± 2.75 c	71.75 ± 1.71 a	88.85 ± 4.37 c	11.42 ± 0.18 b	5.18 ± 0.55 c	106.50 ± 8.27 a

Note: The results are shown as the mean ± SE for three biological replicates (for each replicate, n = 6). Means denoted by the same letters were not significantly different at *p* < 0.05 according to Tukey’s test.

**Table 2:** Effects of storage temperature on the amino acid composition and contents (g kg^−1^) in fruits of three pepper cultivars.

	P1632			P1833			P1622		
	Control	20 °C-48 h	30 °C-48 h	Control	20 °C-48 h	30 °C-48 h	Control	20 °C-48 h	30 °C-48 h
Total amino acids (T)	48.91 ± 2.82 a	38.07 ± 2.25 c	43.05 ± 2.42 b	44.39 ± 2.81 a	42.23 ± 2.51 b	31.97 ± 1.90 b	47.97 ± 2.55 a	43.85 ± 2.28 b	43.71 ± 2.15 b
Essential amino acids (A)	20.35 ± 1.27 a	14.99 ± 0.98 c	17.32 ± 1.11 b	18.29 ± 1.18 a	16.93 ± 1.06 b	14.00 ± 0.86 c	20.09 ± 0.97 a	17.91 ± 0.85 b	18.11 ± 0.81 b
Non-essential amino acids (B)	28.55 ± 1.55 a	23.08 ± 1.27 c	25.73 ± 1.31 b	26.10 ± 1.63 a	25.29 ± 1.45 a	17.97 ± 1.04 b	27.88 ± 1.58 a	25.94 ± 1.43 b	25.60 ± 1.34 b
Children’s essential amino acids (C)	4.09 ± 0.16 a	3.28 ± 0.14 c	3.48 ± 0.17 b	3.76 ± 0.21 a	3.61 ± 0.22 a	2.77 ± 0.14 b	4.19 ± 0.20 a	3.87 ± 0.24 b	3.88 ± 0.17 b
Tasty amino acids (D)	9.01 ± 0.58 a	7.18 ± 0.49 c	8.44 ± 0.51 b	8.11 ± 0.59 a	8.02 ± 0.55 a	5.18 ± 0.33 b	7.90 ± 0.53 a	8.06 ± 0.52 a	7.46 ± 0.46 b
Sweet amino acids (E)	12.45 ± 0.73 a	10.05 ± 0.52 c	11.17 ± 0.50 b	11.47 ± 0.68 a	10.44 ± 0.54 b	8.60 ± 0.51 c	12.63 ± 0.66 a	11.17 ± 0.52 b	11.34 ± 0.54 b
Aromatic amino acids (F)	5.39 ± 0.26 a	3.23 ± 0.17 c	4.49 ± 0.25 b	4.77 ± 0.26 a	4.71 ± 0.23 a	3.02 ± 0.15 b	5.47 ± 0.26 a	4.81 ± 0.28 b	4.86 ± 0.24 b
A/T %	41.62 ± 2.60 a	39.37 ± 2.57 b	40.23 ± 2.58 b	41.20 ± 2.66 b	40.10 ± 2.51 c	43.80 ± 2.69 a	41.87 ± 2.02 a	40.85 ± 1.94 c	41.44 ± 1.85 b
B/T %	58.38 ± 3.17 b	60.63 ± 3.34 a	59.77 ± 3.04 a	58.80 ± 3.67 a	59.90 ± 3.43 a	56.20 ± 3.25 b	58.13 ± 3.29 a	59.15 ± 3.24 a	58.56 ± 3.07 a
C/T %	8.36 ± 0.33 ab	8.60 ± 0.37 a	8.08 ± 0.39 b	8.46 ± 0.47 a	8.56 ± 0.52 a	8.66 ± 0.44 a	8.73 ± 0.42 a	8.82 ± 0.55 a	8.87 ± 0.39 a
D/T %	18.41 ± 1.19 b	18.85 ± 1.29 b	19.60 ± 1.18 a	18.26 ± 1.33 a	18.99 ± 1.30 a	16.20 ± 1.03 b	16.47 ± 1.10 b	18.39 ± 1.19 a	17.06 ± 1.05 b
E/T %	25.46 ± 1.49 b	26.40 ± 1.37 a	25.94 ± 1.16 ab	25.84 ± 1.53 b	24.73 ± 1.28 c	26.89 ± 1.60 a	26.33 ± 1.38 a	25.46 ± 1.19 a	25.93 ± 1.24 a
F/T %	11.01 ± 0.53 a	8.49 ± 0.45 c	10.43 ± 0.58 b	10.74 ± 0.59 b	11.17 ± 0.54 a	9.44 ± 0.47 c	11.41 ± 0.54 a	10.98 ± 0.64 b	11.12 ± 0.55 b

Note: T, total amino acids; A, essential amino acids; B, non-essential amino acids; C, children’s essential amino acids; D, tasty amino acids; E, sweet amino acids; F, aromatic amino acids. The results are shown as the mean ± SE for three biological replicates (for each replicate, n = 6). Means denoted by the same letters were not significantly different at *p* < 0.05 according to Tukey’s test.

**Table 3:** Effects of storage temperature on the essential amino acid scores of three pepper cultivars.

Essential	Amino Acid Pattern Spectrum	P1632			P1833			P1622		
Amino Acid		Control	20 °C-48 h	30 °C-48 h	Control	20 °C-48 h	30 °C-48 h	Control	20 °C-48 h	30 °C-48 h
Thr	2.30	5.59 ± 0.31 a	5.70 ± 0.37 a	5.40 ± 0.28 b	5.57 ± 0.38 a	4.65 ± 0.28 b	5.83 ± 0.38 a	5.77 ± 38 a	5.52 ± 0.30 a	5.55 ± 0.25 a
Val	3.90	5.42 ± 0.27 a	5.44 ± 0.32 a	4.98 ± 0.28 b	5.25 ± 0.20 b	5.25 ± 0.24 b	6.02 ± 0.28 a	5.25 ± 0.23 a	5.20 ± 0.21 a	5.36 ± 0.27 a
Ile	3.00	4.49 ± 0.31 a	4.48 ± 0.29 a	4.16 ± 0.23 b	4.32 ± 0.25 a	4.27 ± 0.26 a	4.49 ± 0.25 a	4.25 ± 0.17 a	4.10 ± 0.23 a	4.28 ± 0.21 a
Leu	5.90	11.00 ± 0.76 a	11.16 ± 0.87 a	10.70 ± 0.88 b	11.00 ± 0.79 a	10.82 ± 0.88 a	10.98 ± 0.72 a	11.14 ± 0.54 a	10.80 ± 0.50 b	11.09 ± 0.43 a
Lys	4.50	7.30 ± 0.51 a	7.53 ± 0.47 a	7.27 ± 0.49 a	7.21 ± 0.54 b	7.31 ± 0.50 b	8.11 ± 0.63 a	7.44 ± 0.40 a	7.28 ± 0.30 a	7.19 ± 0.30 a
Met + Cys	2.20	2.00 ± 0.12 a	2.07 ± 0.13 a	1.96 ± 0.14 a	2.11 ± 0.11 b	2.06 ± 0.09 b	2.59 ± 0.13 a	2.08 ± 0.08 b	2.22 ± 0.11 a	2.25 ± 0.11 a
Phe + Tyr	3.80	11.01 ± 0.53 a	8.49 ± 0.45 c	10.43 ± 0.58 b	10.74 ± 0.59 b	11.17 ± 0.54 a	9.44 ± 0.47 c	11.41 ± 0.54 a	10.98 ± 0.64 b	11.12 ± 0.55 ab

Note: Amino acids are represented by the 3-letter abbreviation code. Tyr, tyrosine; Val, valine; Ile, isoleucine; Leu, leucine; Lys, lysine; Met, methionine; Cys, cysteine; Phe, phenylalanine; Tyr, tyrosine. The results are shown as the mean ± SE for three biological replicates (for each replicate, n = 6). Means denoted by the same letters were not significantly different at *p* < 0.05 according to Tukey’s test.

## Data Availability

The data that support the findings of this study are available from the corresponding author upon reasonable request. Data is not publicly available due to privacy restrictions.

## Supplementary Materials

The following supporting information can be downloaded at: https://www.mdpi.com/article/10.3390/metabo13070820/s1, Table S1. Effects of temperature on the individual amino acid contents (g kg^−1^) in fruits of three pepper cultivars.
