# Supplementary material for: Effects of Storage Temperature at the Early Postharvest Stage on the Firmness, Bioactive Substances, and Amino Acid Compositions of Chili Pepper (Capsicum annuum L.)
Source: Metabolites. 2023 Jul 5;13(7):820. doi: 10.3390/metabo13070820 (PMC10385654; doi:10.3390/metabo13070820)
Supplement: Supplementary file 1 [file metabolites-13-00820-s001.zip › metabolites-2471602-supplementary.pdf]

Table S1. Effect of temperature on the individual amino acid contents (g kg^{-1}) in fruits of three pepper cultivars.

	P1632			P1833			P1622		
	Control	20°C-48h	30°C-48h	Control	20°C-48h	30°C-48h	Control	20°C-48h	30°C-48h
Aspartic acid	3.21	2.51	3.34	2.88	2.90	1.50	2.55	2.94	2.33
(Asp)[BD]	±0.21 a	±0.18 b	±0.17 a	±0.18 a	±0.17 a	±0.11 b	±0.14 b	±0.11 a	±0.13 c
Threonine (Thr)[AE]	2.73	2.17	2.33	2.47	1.96	1.86	2.77	2.42	2.43
	±0.15 a	±0.14 c	±0.12 b	±0.17 a	±0.12 b	±0.12 b	±0.18 a	±0.13 b	±0.11 b
Serine (Ser)[BE]	3.10	2.56	2.89	2.75	2.62	2.24	3.07	2.72	2.76
	±0.21 a	±0.11 c	±0.12 b	±0.19 a	±0.13 a	±0.14 b	±0.19 a	±0.12 b	±0.11 b
Glutamic acid	5.80	4.66	5.10	5.22	5.12	3.68	5.35	5.13	5.13
(Glu)[BD]	±0.37 a	±0.31 c	±0.34 b	±0.41 a	±0.38 a	±0.22 b	±0.39 a	±0.41 a	±0.33 a
Proline (Pro)[B]	3.20	2.65	2.96	3.03	2.89	2.12	3.31	2.96	2.99
	±0.13 a	±0.14 c	±0.12 b	±0.23 a	±0.14 a	±0.13 b	±0.22 a	±0.13 b	±0.16 b
Glycine (Gly)[BE]	3.19	2.57	2.90	3.03	2.83	2.15	3.31	2.94	3.01
	±0.14 a	±0.11 c	±0.11 b	±0.13 a	±0.14 b	±0.13 c	±0.14 a	±0.14 b	±0.17 b
Alanine (Ala)[BE]	3.43	2.75	3.06	3.22	3.02	2.35	3.48	3.09	3.14
	±0.23 a	±0.16 c	±0.15 b	±0.19 a	±0.15 a	±0.12 b	±0.15 a	±0.13 b	±0.15 b
Valine (Val)[A]	2.65	2.07	2.14	2.33	2.22	1.92	2.52	2.28	2.34
	±0.13 a	±0.12 b	±0.12 b	±0.09 a	±0.10 a	±0.09 b	±0.11 a	±0.09 b	±0.12 b
Methionine (Met)[A]	0.78	0.62	0.66	0.73	0.65	0.63	0.80	0.74	0.75
	±0.05 a	±0.04 c	±0.04 c	±0.04 a	±0.03 b	±0.03 b	±0.03 a	±0.03 b	±0.04 b
Isoleucine (Ile)[A]	2.20	1.71	1.79	1.92	1.80	1.43	2.04	1.80	1.87
	±0.15 a	±0.11 b	±0.10 b	±0.11 a	±0.11 b	±0.08 c	±0.08 a	±0.10 b	±0.09 b
Leucine (Leu)[A]	5.38	4.25	4.61	4.88	4.57	3.51	5.34	4.73	4.85
	±0.37 a	±0.33 c	±0.38 b	±0.35 a	±0.37 a	±0.23 b	±0.26 a	±0.22 b	±0.19 b
Tyrosine (Tyr)[BF]	2.34	1.92	1.84	2.00	2.07	0.97	2.42	2.06	2.12
	±0.09 a	±0.11 b	±0.11 b	±0.08 a	±0.11 a	±0.04 b	±0.14 a	±0.13 b	±0.11 b
Phenylalanine	3.05	1.31	2.66	2.76	2.65	2.05	3.06	2.75	2.74
(Phe)[AF]	±0.17 a	±0.06 c	±0.14 b	±0.18 a	±0.12 a	±0.11 b	±0.12 a	±0.15 b	±0.13 b
Histidine (His)[AC]	1.04	0.89	1.07	1.04	1.01	0.62	1.23	1.22	1.17
	±0.04 a	±0.03 b	±0.04 a	±0.05 a	±0.06 a	±0.02 b	±0.09 a	±0.07 a	±0.05 a
Lysine (Lys)[A]	3.57	2.87	3.13	3.20	3.09	2.59	3.57	3.19	3.14
	±0.25 a	±0.18 c	±0.21 b	±0.24 a	±0.21 a	±0.20 b	±0.19 a	±0.13 b	±0.13 b
Arginine (Arg)[BC]	3.04	2.38	2.41	2.72	2.60	2.15	2.96	2.65	2.71
	±0.12 a	±0.11 b	±0.13 b	±0.16 a	±0.16 a	±0.12 b	±0.11 a	±0.17 b	±0.12 b
Cystine (Cys)[B]	0.20	0.17	0.18	0.21	0.22	0.19	0.20	0.24	0.24
	±0.01 a	±0.01 b	±0.02 b	±0.01 a	±0.01 a	±0.01 b	±0.01 b	±0.02 a	±0.01 a

Note: A, Essential amino acid; B, Non-essential amino acid; C, Children essential amino acid; D, Monosodium glutamate-like amino acid; E, Sweet amino acid; F, Aromatic amino acid. The results are shown as the mean ± SE of triplicate samples. Means denoted by the same letter did not differ significantly at $p < 0.05$ according to Tukey's test.
